# Association Between Redox and Inflammatory Biomarkers with the Presence and Severity of Obstructive Sleep Apnea

**DOI:** 10.3390/medicina61091557

**Published:** 2025-08-29

**Authors:** Ana Ninić, Branislava Rajkov, Jelena Kotur-Stevuljević, Sanja Erceg, Miron Sopić, Jelena Munjas, Vesna Spasojević-Kalimanovska, Marija Mitrović, Lidija Memon, Vera Gardijan, Milica Brajković, Slobodan Klašnja, Marija Zdravković

**Affiliations:** 1 Department of Medical Biochemistry, Faculty of Pharmacy, University of Belgrade, 11000 Belgrade, Serbia; aninic@pharmacy.bg.ac.rs (A.N.); branislavasupljeglav@gmail.com (B.R.); jkotur@pharmacy.bg.ac.rs (J.K.-S.); miron.sopic@pharmacy.bg.ac.rs (M.S.); jelenaj@pharmacy.bg.ac.rs (J.M.); vkalima@pharmacy.bg.ac.rs (V.S.-K.); marijamihajlovic89@yahoo.com (M.M.); 2 Department of Laboratory Diagnostics, University Medical Center Bežanijska kosa, 11000 Belgrade, Serbia; lidijamemon13@gmail.com; 3 Department of Pulmology, University Medical Center Bežanijska kosa, 11000 Belgrade, Serbia; vgardijan@gmail.com (V.G.); brajkovic.milica77@gmail.com (M.B.); 4 Department of Cardiology, University Medical Center Bežanijska kosa, 11000 Belgrade, Serbia; slobodan.klasnja@gmail.com (S.K.); sekcija.kardioloska@gmail.com (M.Z.); 5 Faculty of Medicine, University of Belgrade, 11000 Belgrade, Serbia

**Keywords:** obstructive sleep apnea, full-length receptor for advanced glycation end products, transforming growth factor beta 1, soluble receptor for advanced glycation end products, total oxidative status, oxidative stress index, total antioxidant status

## Abstract

*Background and Objectives:* Obstructive sleep apnea (OSA) represents an increasing public health concern, closely linked with cardiovascular, metabolic, and neurocognitive disorders, as well as impaired quality of life. The complex pathophysiology of OSA involves upper airway dysfunction, oxidative stress, and inflammation, with endothelial dysfunction considered central to its associated comorbidities. Despite notable advances in OSA research, the biological mechanisms driving these complications remain insufficiently understood. The present study aimed to examine the associations between redox status, proinflammatory biomarkers, and the gene expression of full-length receptor for advanced glycation end products (flRAGE) and transforming growth factor beta 1 (TGF-β1) in relation to the presence and severity of OSA. *Materials and Methods:* The study cohort comprised 125 participants with diagnosed OSA and 42 controls without evidence of OSA. General and clinical characteristics were recorded for all participants. Laboratory analyses included the assessment of redox and inflammatory markers in serum and plasma, while flRAGE and TGF-β1 messenger ribonucleic acids (mRNA) were quantified in peripheral blood mononuclear cells. *Results:* Patients with OSA demonstrated elevated oxidative stress and inflammation, characterized by increased total antioxidant status (TAS) and C-reactive protein CRP levels, together with reduced concentrations of soluble RAGE (sRAGE). The severity of OSA, indicated by the apnea-hypopnea index, increases total oxidative status (TOS) and TGF-β1 mRNA, while sRAGE decreases. The sRAGE–ROS-related factor was negatively associated with OSA, whereas the redox status factor showed a positive association. TOS was independently and positively correlated with OSA severity. *Conclusions:* Individuals with OSA exhibit a state of enhanced oxidative stress and inflammation. Increasing severity of OSA was associated with rising TOS and TGF-β1 mRNA expression, accompanied by declining sRAGE concentrations. A combined redox–inflammatory biomarker profile was found to be associated with both the presence and severity of OSA.

## 1. Introduction

Obstructive sleep apnea (OSA) is a prevalent sleep disorder characterized by recurrent episodes of partial or complete upper airway obstruction during sleep, resulting in intermittent hypoxia and disruption of normal sleep architecture [1]. Hallmark features of OSA include repeated episodes of apnea (complete cessation of airflow) and hypopnea (partial reduction in airflow) during sleep. They collectively contribute to chronic intermittent hypoxaemia (CIH), hypercapnia, heightened sympathetic activity, and pronounced oxidative stress accompanied by systemic inflammation [1].

OSA represents a major public health concern, with a high and rising prevalence among adults [2]. It is strongly associated with cardiovascular and cerebrovascular disease [3], metabolic dysfunction, neurocognitive impairment, accelerated ageing, and diminished quality of life [4]. The pathophysiology of OSA reflects a complex interaction between anatomical predisposition and neuromuscular dysfunction of the upper airway, together with oxidative stress and inflammation. However, the precise mechanisms linking these processes remain incompletely understood [1]. It is postulated that endothelial dysfunction, driven by these pathophysiological pathways, plays a central role in the development of OSA-related comorbidities [3].

The severity of OSA is defined by the frequency of apneic and hypopneic events per hour, as assessed by polysomnography [5]. Current therapeutic approaches span lifestyle modification and continuous positive airway pressure therapy, through to surgical interventions and emerging molecular-based treatments [5].

Fluctuating oxygen concentrations, arising from alternating periods of hypoxia and reoxygenation, promote the excessive generation of reactive oxygen species (ROS) [6]. These highly reactive molecules can damage critical cellular components, including proteins, lipids, and nucleic acids. The recurrent cycles of oxygen deprivation and restoration disturb redox homeostasis, leading to widespread cellular and organ injury that disrupts metabolic regulation [7]. Several biomarkers of redox status, including total oxidative status (TOS) [8], thiobarbituric acid reactive substances (TBARS) [9], total antioxidant status (TAS) [8], and advanced oxidation protein products (AOPP) [9], have been implicated in oxidative stress among individuals with OSA.

Intermittent oxygen deprivation also provokes the release of proinflammatory mediators [6]. Systemic inflammation is a hallmark of OSA and represents a pivotal mechanism in the development of cardiovascular and metabolic disorders. Individuals with OSA frequently exhibit elevated circulating levels of proinflammatory biomarkers, including C-reactive protein (CRP) and cytokines such as tumour necrosis factor-alpha and interleukin-6. This heightened inflammatory activity impairs endothelial function and accelerates the onset and progression of OSA-related complications [10].

The membrane-bound full-length receptor for advanced glycation end products (flRAGE) and its soluble isoform (sRAGE) are recognized as key mediators of inflammatory and oxidative stress pathways [11]. Binding of ligands to flRAGE activates nuclear factor kappa B (NF-κB), thereby enhancing oxidative stress and stimulating the production of proinflammatory mediators [11]. Once activated, NF-κB further promotes ligand–flRAGE interactions, perpetuating a cycle of cytokine and tissue factor expression [11,12]. The activation of flRAGE has been implicated in the pathogenesis and progression of a range of diseases, including OSA, cancer, diabetes mellitus, cardiovascular disease, and osteoarthritis [13].

In contrast to flRAGE, sRAGE lacks intracellular signalling domains and is therefore unable to initiate downstream cellular responses. Instead, sRAGE is thought to mitigate ligand-induced oxidative and inflammatory effects by functioning as a decoy receptor [13]. Owing to this property, numerous studies have investigated its potential anti-inflammatory and antioxidant roles, suggesting that sRAGE may contribute to lowering the risk of cardiovascular disease and other OSA-related comorbidities [13].

Transforming growth factor-beta 1 (TGF-β1) is a multifunctional cytokine that plays a central role in numerous biological processes, including embryonic development, immune regulation, wound healing, angiogenesis, fibroblast activation, and the maintenance of cellular homeostasis between proliferation and apoptosis [14]. Dysregulation of TGF-β1 is implicated in a range of pathological conditions, including inflammatory diseases, fibrosis, infections, and tumour development [14]. It is associated with enhanced fibrogenic activity in organs such as the lungs, liver, kidneys, and heart [14]. TGF-β1 activates several intracellular signalling pathways, notably the Ras-extracellular signal-regulated kinase 1/2 and NF-κB cascades [14], which are likewise triggered by AGE–flRAGE interactions [12].

flRAGE and TGF-β1 interact through complex signalling networks that are not yet fully elucidated. They modulate each other’s activity while also acting independently on distinct molecular targets [13,14,15]. This interplay influences oxidative stress, extracellular matrix remodelling, and cytokine production, thereby contributing to the development and progression of complications associated with OSA [15].

Despite considerable advances in understanding OSA, the precise biological mechanisms underlying its associated complications remain incompletely elucidated. Deciphering these complex pathways and identifying novel biomarkers may enable the development of more targeted and effective therapeutic strategies. In this context, the present study aimed to investigate the associations between redox status, proinflammatory biomarkers, and gene expression profiles with both the presence and severity of OSA. By applying statistical approaches such as principal component analysis (PCA) and logistic regression, we sought to identify key predictive factors for OSA and gain further insight into the potential molecular mechanisms driving the condition. 

## 2. Materials and Methods

### 2.1. Study Participants

Between November 2016 and December 2017, we conducted a cross-sectional study involving adult patients referred for polysomnography at the University Hospital Medical Centre Bežanijska kosa in Belgrade, within the Pulmonology Department. The study population comprised male and female patients aged 20 years or older, who exhibited a suspected diagnosis of OSA and provided informed consent to participate. Patients presenting with central sleep apnea, inherited metabolic disorders, or currently using antidepressants and/or sedatives at the time of screening were excluded from the study. A total of 167 participants met the eligibility criteria; of these, 125 were diagnosed with OSA based on polysomnography results, while 42 participants showed no evidence of the disorder and were assigned to the control group.

The study received ethical approval from the Ethics Committees of the Hospital Medical Centre Bežanijska kosa (No. 3747/4) and the University of Belgrade-Faculty of Pharmacy (No. 1074/2) and was conducted in accordance with the ethical principles outlined in the Declaration of Helsinki. Following informed consent, data were collected regarding participants’ age, height, weight, systolic and diastolic blood pressure, the presence of comorbid type 2 diabetes, smoking status, alcohol consumption, physical activity and current use of antihypertensive and antihyperlipidemic therapies. The body mass index (BMI) was calculated using the measured height and weight data, applying the formula weight (kg) divided by height squared (m^2^).

### 2.2. Sleep Study

Participants underwent overnight polysomnography from 22:00 to 06:00 h. The testing was conducted using the Alice PDx device (Philips Respironics, Inc., Murrysville, PA, USA) within a sleep laboratory setting at the Pulmonology Department of the Hospital Medical Centre Bežanijska Kosa. On the morning following the testing, each recording was analyzed by a trained specialist at 09:00 h, and data were collected on the apnea–hypopnea index (AHI), oxygen desaturation index (ODI), and average and minimal oxygen saturation (SaO_2_). The AHI was defined as the number of apnea and hypopnea episodes recorded per hour of sleep. A diagnosis of OSA was established for individuals with an AHI greater than 5. The severity of OSA was categorized as follows: mild (AHI > 5 and <14), moderate (AHI ≥ 15 and <30), and severe (AHI ≥ 30). The ODI was calculated as the number of oxygen saturation drops of ≥3% from baseline per hour of sleep. Participants with an AHI of 5/6 and an ODI < 2 were not classified as having OSA.

### 2.3. Laboratory Measurements

Venous blood samples were obtained using standard venepuncture techniques following polysomnography studies and a 12-h fasting period. Biochemical parameters were analyzed in the collected serum samples using the Roche Cobas 6000 analyzer (Roche Diagnostics International Ltd., Rotkreuz, Switzerland). The analyses included glucose, total protein, albumin, total bilirubin, direct bilirubin, CRP, urea, creatinine, and uric acid.

Redox status markers were measured in serum samples, except for the superoxide anion radical (O_2_^.−^), which was measured in plasma samples. The generation of O_2_^.−^ was assessed using the Auclair and Voisin method, which measures the reduction rate of the nitroblue tetrazolium reagent [16]. The intra- and inter-assay coefficients of variation (CV) for O_2_^.−^ were 3.46% and 5.04%, respectively. AOPPs were measured using the potassium iodide/glacial acetic acid-based method described by Witko-Sarsat [17], with intra- and inter-assay CVs of 1.15% and 4.09%, respectively. The total concentration of protein sulphydryl (tSH) groups was determined using a modified version of Ellman’s spectrophotometric method, which relies on the reaction of 2,2′-dinitro-5,5′-dithiobenzoic acid with aliphatic thiol compounds in an alkaline medium [18]. The intra- and inter-assay CVs for tSH groups were 2.90% and 4.67%, respectively. Superoxide dismutase (SOD) activity was measured using the method described by Misra and Fridovich, which is based on the enzyme’s inhibition of epinephrine auto-oxidation in an alkaline bicarbonate buffer [19]. The intra- and inter-assay CVs for SOD activity were 4.78% and 8.11%, respectively. The concentration of TBARS was measured using the Girotti method [20], with intra- and inter-assay CVs of 1.40% and 3.80%, respectively. Pro-oxidant-antioxidant balance (PAB) levels were determined using an assay based on 3,3′,5,5′-tetramethylbenzidine and its cation as redox indicators [21]. The intra- and inter-assay CVs for PAB were 2.74% and 4.11%, respectively.

Ischaemia-modified albumin (IMA) was quantified using a colorimetric assay based on the reduced binding capacity of exogenous cobalt to the N-terminus of serum albumin [22]. The intra- and inter-assay CVs for IMA were 3.26% and 5.08%, respectively. TOS was determined using the optimized method described by Erel, in which oxidants present in the sample convert ferrous ion–o-dianisidine complexes to ferric ions, which subsequently form a colored complex with xylenol orange [23]. The intra- and inter-assay CVs for TOS were 2.70% and 4.60%, respectively. TAS was measured using a colorimetric method employing a novel radical cation, as refined by Erel [24]. The intra- and inter-assay CVs for TAS were 3.05% and 4.43%, respectively. The oxidative stress index (OSI) was calculated according to the formula TOS/TAS × 100.

The ILAB 650 analyzer (Instrumentation Laboratory, Milan, Italy) was employed to measure O_2_^.−^, AOPPs, SOD, PAB, TOS, and TAS. The SPECTROstar Nano microplate reader (BMG Labtech, Ortenberg, Germany) was used to assess tSH groups, TBARS, and IMA. Plasma concentrations of sRAGE were quantified using enzyme-linked immunosorbent assays (DuoSet, R&D Systems, Wiesbaden, Germany), according to the manufacturer’s instructions. The intra- and inter-assay CVs for sRAGE were 2.50% and 8.60%, respectively.

Following plasma separation, peripheral blood mononuclear cells (PBMCs) were isolated by the Ficoll-Paque^®^ PLUS gradient-gel (GE Healthcare, Chicago, IL, USA) per the manufacturer’s instructions and were suspended in 1 mL of TRIzol^TM^ reagent (Invitrogen Life Technologies, Foster City, CA, USA) before freezing at −80 °C until the ribonucleic acid (RNA) isolation. The modified TRIzol^TM^-chloroform separation technique was used to isolate the total RNA from PBMCs [25]. Reverse transcription and quantitative polymerase chain reaction techniques were carried out on the 7500 real-time PCR System using TaqMan^TM^-based gene expression assays (Applied Biosystems, Foster City, CA, USA) for TGF-β1 (Hs00998133_m1) and flRAGE (Hs00153957_m1) genes. Gene expression was quantified using the relative standard curve method. The relative expression levels of the target genes were determined by calculating the ratio of each target gene’s messenger RNA (mRNA) and the constitutively expressed housekeeping gene, β-actin mRNA. The following equations were used: normalized flRAGE mRNA levels = flRAGE mRNA/β-actin mRNA and normalized TGF-β1 mRNA levels = TGF-β1 mRNA/β-actin mRNA.

### 2.4. Statistical Analysis

Statistical analyses were conducted using SPSS Statistics v.29 (IBM, Chicago, IL, USA). Data distribution was assessed with the Kolmogorov–Smirnov and Shapiro–Wilk tests. Normally distributed data were expressed as mean ± standard deviation and compared using the Student’s *t*-test or one-way ANOVA with Tukey’s post hoc test. Non-normally distributed data were presented as median (interquartile range) and compared using the Mann–Whitney U test or Kruskal–Wallis test. Categorical variables were expressed as absolute and relative frequencies and analyzed using the Chi-square test for contingency tables. Redox status, inflammatory, and gene expression biomarkers were adjusted for age, sex, BMI, antihypertensive and antihyperlipidemic therapies, type 2 diabetes, CRP, and uric acid using predictive values from a regression model to determine whether these covariates influenced group differences. Prior to adjustment, multicollinearity was assessed, and all variables exhibited a variance inflation factor (VIF) ≤ 2.0. Adjusted data were presented as adjusted mean ± 95% confidence interval (CI) for normally distributed variables and as median (interquartile range) for skewed variables. Spearman correlation analysis was performed to examine correlations between polysomnography parameters and the tested biomarkers. In-depth associations were evaluated using univariate and multivariate binary and ordinal logistic regression analyses. The dependent variables were OSA presence (0 = no OSA; 1 = OSA) and OSA severity (1 = mild, 2 = moderate, 3 = severe), while the independent variables were implicated in OSA and exhibiting no multicollinearity (VIF < 2.0). Logistic regression results were reported as odds ratios (ORs) with 95% CIs. Finally, PCA with varimax rotation was performed to reduce the number of redox and proinflammatory biomarkers to a smaller set of factors capturing similar variance. Factors were extracted based on Eigenvalues >1, and variables with factor loadings > 0.5 were retained. Factor scores were subsequently used as independent variables in binary and ordinal logistic regression analyses to identify significant predictors of OSA presence and severity. Statistical significance was set at *p* < 0.05.

## 3. Results

### 3.1. Control Group vs. Obstructive Sleep Apnea

#### 3.1.1. Demographic, Clinical, and Biochemical Data Examination

General data of the study population are given in Table 1. Men and women were evenly distributed between the tested groups. Patients with OSA were older. They had higher BMI, systolic blood pressure, glucose, CRP, urea, and uric acid levels. Expectedly, all data obtained from polysomnograph (AHI, ODI, minimal and average SaO_2_) were higher in OSA compared to non-OSA patients. The prevalence of antihypertensive users was higher in OSA patients.

#### 3.1.2. Oxidative Stress Biomarkers Examination

Regarding the redox status markers, they did not differ between groups except for TAS being higher and sRAGE being lower in OSA patients compared to non-OSA patients (Table 2).

#### 3.1.3. Correlation Analysis

Furthermore, we analyzed the correlation between biochemical and redox status biomarkers with polysomnography data (Table 3). AHI and ODI were correlated positively with age, BMI, glucose, CRP, urea, uric acid, AOPP, and TOS. Only AHI was correlated positively with OSI and negatively with sRAGE. Average and minimal SaO_2_ were correlated negatively with age, BMI, glucose, urea, CRP, uric acid, AOPP, TOS, and TAS and positively with O_2_^.−^, sRAGE, and normalized flRAGE mRNA. Only minimal SaO_2_ correlated negatively with total proteins and OSI.

#### 3.1.4. Regression and Principal Component Analyses

To determine the predictive abilities of the redox status biomarkers (AOPP, TOS, OSI, and sRAGE), in relation to the presence of OSA, we performed a binary logistic regression analysis. These markers correlated significantly with the AHI in the Spearman correlation analysis (Table 3). None of them was a significant predictor for the presence of OSA. 

PCA was applied to redox status, gene expression, and proinflammatory biomarkers to determine their relationship with OSA. The adequacy of the sample was confirmed using the Keiser–Meier–Olkin measure index equal to 0.674. Bartlett’s test of sphericity was significant (*p* < 0.001). The factors were given in Table 4.

This PCA extracted four significant factors with a total percentage of explainable variation of 62% of the analysed biomarkers. The first factor (redox status-related factor) explained 27% of the total variance in the analyzed population. It was associated with positive loadings of the TOS, SOD, AOPP, IMA, and tSH groups and with negative loadings of TAS. The second factor (proinflammatory gene-related factor) explained 13% of the total variance in the population studied. It was associated with a positive loading of normalized TGF-β1 and RAGE mRNAs. The third factor (sRAGE-ROS-related factor) explained 11% of the total variance in the analyzed population. It was associated with positive loading of sRAGE and O_2_^.–^. The fourth factor (pro-oxidant-related factor) explained 11% of the total variance in the population studied. It was associated with a positive loading of PAB and TBARS.

Using the scores derived from the PCA, we performed an univariate binary logistic regression analysis to determine which factor was associated with OSA status (Table 5). Significant predictive power was found for only one factor, the sRAGE-ROS-related factor for the presence of OSA (OR = 0.568, *p* = 0.009). Lower values of the redox status-related factor were associated with a 43.2% higher probability of OSA.

### 3.2. Examination in Patients with Obstructive Sleep Apnea

#### 3.2.1. Demographic, Clinical, and Biochemistry Data According to the Apnea–Hypopnea Index

Our further aim was to analyze clinical biomarkers and the redox status in OSA patients only. We formed three groups according to the severity of OSA. The general data of OSA patients are shown in Table 6. Patients with severe OSA had a higher BMI and higher uric acid levels than patients with mild and moderate forms of the disease. Glucose and creatinine levels were higher in patients with severe OSA than in those with mild OSA. As expected, all polysomnographic data differed significantly between all three study groups.

#### 3.2.2. Redox Status and Inflammatory Biomarkers According to the Apnea–Hypopnea Index

Redox status markers of the study groups are given in Table 7. AOPP, tSH groups, and OSI were higher while sRAGE was lower in patients with severe OSA compared to those with mild and moderate forms of disease. TOS was higher in patients with moderate and severe OSA than in the mild form of the disease. Normalized TGF-β1 mRNA was higher in severe OSA than in moderate OSA.

#### 3.2.3. Correlation Analysis in Patients with Obstructive Sleep Apnea

Spearman correlation analysis showed positive correlations of AHI and ODI with BMI, glucose, uric acid, AOPP, total SH groups, IMA, TOS and OSI and negative correlations with sRAGE. In addition, only ODI correlated positively with CRP and negatively with total and direct bilirubin. Average and minimal SaO_2_ correlated positively with BMI and sRAGE and negatively with total proteins, CRP, uric acid, and AOPP. In addition, only average SaO_2_ correlated positively with normalized RAGE mRNA, while only minimal SaO_2_ correlated negatively with glucose, TOS, and OSI (Table 8).

#### 3.2.4. Regression and Principal Component Analyses in Patients with Obstructive Sleep Apnea

To determine which redox status marker is significantly associated with the OSA degree, we performed an ordinal logistic regression analysis. The significant predictors are listed in Table 9. The results showed that the TOS, OSI, AOPP, IMA, and tSH groups were positively associated with a higher OSA degree. Whereas, sRAGE was negatively associated with a higher OSA degree. When significant predictors from the univariate ordinal logistic regression were included in the models with covariates significantly correlated with AHI from Table 8 (BMI and uric acid), type 2 diabetes, smoking status, and therapies, the independent predictor of higher OSA degree was only TOS (OR = 1.037, *p* = 0.034). An increase in TOS was associated with a 3.7% higher probability of a higher OSA degree.

Using the scores derived from the PCA, we performed a univariate ordinal logistic regression analysis to determine which factor is associated with a higher degree of OSA. Significant predictive ability was demonstrated for only one factor, the redox status-related factor (OR = 1.598, *p* = 0.029). Elevated levels of the redox status-related factor were associated with a 59.8% higher probability of higher OSA severity. The results of these analyses are shown in Table 10.

## 4. Discussion

OSA is a multifactorial disorder whose onset and progression are influenced by a wide range of risk factors, including age, sex, family history, craniofacial anatomy, and other structural characteristics of the upper respiratory tract, as well as alcohol and tobacco use [1,2]. Among these, obesity represents one of the most significant determinants of OSA risk [2]. Oxidative stress and inflammation, both closely linked to obesity, are thought to play pivotal roles in the pathophysiology of OSA and its associated complications, including cardiovascular and cerebrovascular disease, metabolic-associated fatty liver disease, and certain malignancies [1,3,6]. ROS can trigger and maintain inflammatory processes, while inflammation further increases oxidative damage. This creates a vicious cycle that promotes endothelial dysfunction and increases cardiovascular risk, as well as contributing to other OSA-related comorbidities.

To our knowledge, this is the first study to simultaneously examine multiple biomarkers of redox balance and inflammatory status in relation to both the presence and severity of OSA. Our findings indicate that alterations in redox and inflammatory homeostasis are evident in patients with OSA. Compared with the control group, OSA patients exhibited higher TAS and lower sRAGE levels. Furthermore, patients with more severe OSA demonstrated increased levels of AOPP, tSH groups, TOS, OSI, and TGF-β1 mRNA, alongside lower sRAGE concentrations, compared with those with milder forms of the disease.

A key regulator in the cycles of oxygen deprivation and reoxygenation that precipitate oxidative stress is hypoxia-inducible factor 1-alpha (HIF-1α). This transcription factor is stabilized under hypoxic conditions and accumulates in the cell, subsequently translocating to the nucleus where it activates the transcription of genes implicated in oxygen metabolism and inflammatory pathways [26,27]. During recurrent hypoxia, sustained nuclear accumulation of HIF-1α amplifies these responses. The resulting increase in ROS production arises from impaired mitochondrial electron transport, activation of inflammatory cells, and O_2_^.−^ generation by xanthine oxidase and nicotinamide adenine dinucleotide phosphate oxidase [6,27].

TOS reflects the overall burden of oxidants, including ROS, within the body, and elevated values are indicative of heightened oxidative stress [23]. In contrast, TAS represents the pool of non-enzymatic endogenous antioxidants that mitigate oxidative damage by neutralising free radicals [24]. As noted above, in OSA, recurrent cycles of hypoxia and reoxygenation promote excessive ROS generation, leading to an increase in TOS. In line with this, Olszewska et al. demonstrated that the uvular mucosa of OSA patients exhibited increased TOS and OSI, accompanied by decreased TAS, thereby underscoring the contribution of oxidative stress to the pathophysiology of OSA [8]. In a study conducted by Kang et al., no significant differences in TOS or TAS were observed between patients with OSA and controls, nor across different OSA severity groups [28]. These findings contrast with our results, where TAS was higher in OSA patients compared with controls (Table 2). Moreover, in severe OSA, tSH groups were elevated relative to mild and moderate cases (Table 7). Taken together, the increased TAS, alongside higher tSH groups concentrations, may reflect a compensatory upregulation of antioxidant defences in response to elevated ROS levels [29]. As a measure of the oxidant-buffering capacity of a sample, TAS reflects compensatory mechanisms aimed at counteracting disturbances in redox balance. An alternative explanation relates to the biochemical composition of TAS, which is largely determined by protein SH groups, uric acid, ascorbic acid, bilirubin, urea, and α-tocopherol [24]. In the present study, urea and uric acid levels were significantly higher in OSA patients compared with controls (Table 1). By contrast, concentrations of total protein, albumin, total bilirubin, and direct bilirubin did not differ significantly between the groups, although a tendency toward higher values was observed. In line with our observations on TAS, a recent study by Ozturk and colleagues also reported increased TAS alongside elevated TOS [30]. They suggested that these apparently contradictory results might be attributable to comorbidities and medication use among OSA patients. In our study, TOS and OSI did not differ significantly between patients and controls (Table 2), but both correlated positively with AHI (Table 3). While TAS was unaffected by OSA severity, TOS and OSI were higher in patients with severe disease compared with those with milder forms of OSA (Table 7). Only TOS was independently and positively associated with OSA severity (Table 9). This association remained significant when incorporated into the composite redox status-related factor, whereby higher values were strongly linked to more severe forms of OSA (Table 10). Collectively, these findings are consistent with the broader body of evidence supporting the concept that OSA is characterized by heightened oxidative stress.

As highly reactive molecules, ROS can interact with and damage critical cellular components, including proteins, lipids, and nucleic acids [6,7]. TBARS, a by-product of lipid peroxidation formed during the degradation of lipid hydroperoxides [20], is frequently employed as a biomarker of oxidative stress in a range of diseases, including OSA. Hu and colleagues [9] reported elevated TBARS levels in OSA patients compared with controls, and Lavie et al. [31] similarly demonstrated that TBARS concentrations were approximately twice as high in OSA patients as in control subjects with cardiovascular disease. By contrast, in the present study, TBARS levels did not differ significantly either between OSA patients and controls or across categories of OSA severity (Table 2 and Table 7).

TBARS levels also showed no correlation with polysomnographic parameters. Even within the PCA-derived Pro-oxidant-related factor, TBARS demonstrated no significant associations with either the presence or severity of OSA (Table 5 and Table 10). These findings suggest that significant lipid peroxidation attributable to oxidative stress was not evident in our study population. This may be explained by the relatively small cohort size or by the high reactivity of TBARS and their potential interactions with various biological molecules [32].

ROS interact with plasma proteins, leading to the formation of dityrosine cross-links and carbonyl derivatives, which subsequently give rise to AOPPs [17]. Elevated circulating AOPP concentrations therefore reflect the degree of oxidative protein damage and serve as a recognized biomarker of oxidative stress. Increased AOPP levels have been reported in OSA patients compared with healthy controls [9,33]. Moreover, elevated AOPP concentrations have been linked to the development of atherosclerosis in OSA, with one study demonstrating significantly higher AOPP levels in OSA patients with arteriosclerosis compared with those without [33]. By contrast, another study found no significant difference in mean AOPP concentrations between OSA patients and controls, although a positive correlation with AHI was observed, indicating that higher AOPP levels are associated with greater OSA severity [34]. These findings are consistent with our results.

Although AOPP levels did not differ significantly between OSA patients and controls, they were markedly higher in patients with severe OSA compared with those with mild or moderate disease (Table 7) and were positively associated with greater OSA severity (Table 9). Similarly, the redox status-related factor, in which AOPP contributed positively alongside TOS, SOD, IMA, and tSH groups, was also positively correlated with OSA severity (Table 10). These findings suggest that protein oxidation, as reflected by AOPP, may be significantly influenced by AHI, potentially contributing to endothelial dysfunction and thereby promoting atherosclerosis and an elevated cardiovascular risk in OSA patients [33]. Moreover, elevated AOPP levels have been identified as an independent risk factor for coronary heart disease [35]. Additionally, AOPPs share several characteristics with AGEs [34], particularly in terms of their formation and biological effects. Both arise as a consequence of oxidative stress, leading to protein modification and cellular damage. AOPPs interact with flRAGE, thereby triggering inflammatory responses. Notably, compared with AGEs, AOPPs elicit an even more pronounced induction of proinflammatory activity, including the upregulation of adhesion molecules and cytokines. The interaction of AGEs with their receptor, flRAGE, is associated with detrimental alterations in the extracellular matrix (ECM), including increased vascular permeability, enhanced contractility, and augmented ECM synthesis. Chronic exposure to AGEs can also provoke maladaptive immune responses, contributing to a range of diabetes-related complications such as nephropathy, impaired wound healing, and heightened susceptibility to infections [11].

FlRAGE activates oxidative stress and inflammatory signalling pathways upon interaction with AGEs, forming a mechanistic basis for the development of OSA-related complications [11,12]. flRAGE expression has been reported to increase proportionally within atherosclerotic plaques with escalating OSA severity [36]. Through flRAGE activation, CIH may contribute to plaque destabilization and promote atherogenesis [36]. Given the CIH-mediated regulation of flRAGE activity and the availability of its substrate, AOPPs, in serum, an upregulation of flRAGE expression in monocytes and lymphocytes might be anticipated. However, in our study, flRAGE mRNA levels were comparable between OSA patients and controls and among patients with different OSA severity (Table 2 and Table 7, respectively). No significant correlations with OSA presence or severity were observed (Table 3 and Table 8, respectively). In view of these unexpected findings, specifically, the absence of significant differences between controls and OSA patients, as well as across OSA severity groups, it is reasonable to consider several potential explanations. One possibility is the relatively modest size of the study cohort. Another factor could be related to population-specific characteristics of the Serbian cohort, which might differ from those reported in other studies. In addition, methodological aspects, particularly the approach used for quantifying flRAGE mRNA expression in PBMCs, may also have influenced the results.

In contrast, sRAGE, which functions as a decoy receptor by neutralizing the effects of AGEs on mitogen-activated protein kinase activation, mitigating oxidative stress, and inhibiting proinflammatory signalling cascades [13,37] was reduced in OSA patients compared with controls (Table 2). sRAGE levels also declined with increasing AHI (Table 3) and were lower in patients with severe OSA than in those with mild or moderate disease (Table 7). Several studies corroborate these findings, reporting decreased plasma sRAGE concentrations in OSA patients [37,38,39]. Furthermore, sRAGE has been shown to correlate negatively with AHI, ODI, and BMI in OSA patients [38], and reduced sRAGE levels have been associated with an elevated risk of cardiovascular disease [40]. Low sRAGE concentrations may reflect its binding to abundant AGEs, such as AOPPs, thereby indicating the presence of systemic inflammation and oxidative stress. Our findings demonstrate that sRAGE levels were negatively associated with OSA degree (Table 9). Similarly, the PCA-derived sRAGE–ROS-related factor, comprising sRAGE and O_2_^.−^, was also negatively correlated with OSA (Table 5), supporting the involvement of reduced sRAGE in the pathophysiology of the disease. Lower values of both sRAGE and the sRAGE–ROS-related factor were indicative of a higher likelihood of more severe OSA.

Reduced sRAGE levels may occur early in the development of hypertensive disease [41]. Given its inverse association with carotid intima–media thickness, sRAGE may play a role in vascular inflammation and the early stages of atherosclerosis [42]. Furthermore, a negative association between sRAGE and advanced stages of hypertension, as well as left ventricular hypertrophy, has been reported in patients receiving antihypertensive therapy [43]. These findings suggest that sRAGE could serve as a valuable prognostic biomarker for cardiac organ damage [41]. Low sRAGE concentrations have also been linked to impaired glucose metabolism in patients with type 2 diabetes and, more broadly, have been associated with poor overall health and increased all-cause mortality [44]. Our results are consistent with these observations. Antihypertensive medications were more frequently used by patients than controls (Table 1). Although the prevalence of type 2 diabetes was not significantly higher among OSA patients or across different OSA severity stages in our cohort, the number of affected individuals appeared to be increasing and approached statistical significance (Table 1 and Table 6). Notably, glucose concentrations were elevated in patients compared with controls (Table 1).

Low-grade inflammation, as indicated by CRP, an established biomarker of inflammation, was observed in participants in our study (Table 1). CRP levels were elevated in OSA patients compared with controls, although differences between patients with varying OSA severity were not statistically significant, despite a clear trend toward higher concentrations. In contrast to sRAGE, CRP correlated positively and significantly with both AHI and ODI (Table 3 and Table 9). This proinflammatory state may be driven by HIF-1α, which enhances the expression of proinflammatory cytokines via activation of the NF-κB signalling pathway [45].

TGF-β1 is a multifunctional cytokine that regulates ECM synthesis and remodelling in various peripheral tissues, including the lung, which can contribute to both fibrogenic and anti-inflammatory processes [46]. Conversely, TGF-β1, produced by regulatory T cells, plays a critical role in suppressing autoreactive T cells, maintaining immune tolerance, and limiting the production of proinflammatory cytokines [14]. Diaz-Garcia and colleagues reported that TGF-β expression in monocytes from OSA patients is upregulated by HIF-1α [47]. They further observed that elevated TGF-β expression may increase the risk of cancer progression and aggressiveness in these individuals. Similarly, Liu et al. demonstrated that OSA enhances TGF-β1 expression in lymphocytes, thereby promoting the maturation and immunosuppressive function of circulating CD4^+^CD25^+^ regulatory T cells [48]. In contrast, TGF-β1 mRNA levels in lymphocytes and monocytes from OSA patients were not upregulated compared with controls and showed no correlation with either AHI or ODI (Table 2 and Table 3, respectively). Nevertheless, expression levels were higher in patients with severe OSA compared with those with moderate disease (Table 7). This increase may reflect direct activation of the TGF-β1 gene by ROS [14]. Similar observations have been reported elsewhere: monocytes from OSA patients display an immunosuppressive profile characterized by elevated TGF-β1 expression induced by HIF-1α. This heightened expression can impair natural killer cell activity, potentially compromising immune defence and increasing susceptibility to infections and malignancies in OSA patients [49]. It is therefore plausible that increased ROS production resulting from CIH in OSA subsequently activates TGF-β1 signalling, contributing to the development of OSA-related comorbidities. Activation of the TGF-β1 signalling pathway contributes to increased vascular resistance and hypertension through vascular remodelling [50]. Via Smad activation, TGF-β1 promotes myofibroblast differentiation and ECM synthesis, ultimately leading to cardiac fibrosis. Elevated TGF-β1 levels are also observed in myocardial infarction, where they exacerbate myocardial injury [51]. Additionally, activation of the Smad–TGF-β1 pathway enhances hepatic glucose synthesis and potentiates the effect of glucagon on gluconeogenesis [52].

Our study results highlight the additional value and potential of the examined oxidative stress and inflammation markers in predicting OSA severity. This was supported by previously published data. As OSA severity increases, TOS rises while total TAS declines, reflecting OSA-related oxidative stress [8,53,54]. OSI not only discriminated between patients with and without OSA [8] but also increased significantly across patients with mild to severe disease [54]. AOPPs may have important clinical relevance in distinguishing patients with more severe OSA from those with milder forms [55]. CRP, an acute-phase protein, may indicate both inflammation and elevated oxidative stress [38,56] and can differentiate patients with more severe OSA from controls [57]. TGF-β1 mRNA and sRAGE may hold greater clinical utility for predicting OSA-related comorbidities [41,42,43,44,47,48,51,52].

Although we did not assess the effects of continuous positive airway pressure therapy on oxidative stress and inflammatory markers in this study, it is likely that such therapy may influence these parameters. Published studies to date have reported conflicting results regarding the impact of continuous positive airway pressure therapy on oxidative stress, inflammation, and OSA-related comorbidities [30,58,59]. Nevertheless, given the serious pathophysiological consequences of OSA linked to oxidative stress, inflammation, and endothelial dysfunction, it is highly recommended that ongoing monitoring of patients receiving this widely used treatment takes place.

This study has several limitations. Firstly, as a cross-sectional study, it identified significant associations between the examined biomarkers, OSA, and OSA severity but causality cannot be inferred. Prospective studies are required to determine whether progressive oxidative stress and inflammation contribute to the development or progression of OSA and its complications. Secondly, the duration of OSA prior to diagnosis in our patients is unknown, leaving it unclear whether the observed biomarker alterations are causal or consequential. Thirdly, future research should assess TGF-β1 protein levels in serum and PBMCs alongside mRNA expression to verify whether protein levels reflect the observed mRNA changes and to further elucidate immunomodulatory dysfunction in these cells. Finally, although the study included a balanced number of male and female participants, a larger cohort, not only from one institution, would strengthen the robustness of our conclusions. Despite these limitations, the present study provides a foundation for future investigations.

## 5. Conclusions

In summary, patients with OSA exhibited a state of heightened oxidative stress and inflammation, characterized by elevated TAS and CRP levels alongside reduced sRAGE. Increasing OSA severity, as reflected by AHI, was associated with higher TOS, OSI, and TGF-β1 mRNA levels, together with lower sRAGE. Furthermore, a composite profile of redox and proinflammatory biomarkers was linked to both the presence and severity of OSA. These findings enhance our understanding of the role of oxidative stress and inflammation in OSA and may have implications for the development of targeted therapeutic strategies.

## Figures and Tables

**Table 1:** Demographic, Clinical, and Laboratory Characteristics of the Studied Groups.

Parameter	Non-OSA Patients N = 42	OSA Patients N = 125	*p*
Male, N (%) ^1^	24 (57.1)	89 (71.2)	0.092
Age, years	51 (35–58)	61 (51–68)	<0.001
BMI, kg/m^2^	27.6 (23.5–30.1)	32.5 (28.3–36.6)	<0.001
SBP, mm Hg	127 (120–135)	134 (126–147)	0.006
DBP, mm Hg	80 (75–90)	85 (80–90)	0.099
AHI, n/h	4.0 (3.1–5.4)	30.4 (17.0–59.0)	<0.001
ODI, n/h	1.6 (9.0–2.9)	30.6 (15.7–64.5)	<0.001
Average SaO_2_, %	96 (95–97)	93 (90–95)	<0.001
Minimal SaO_2_, %	91 (89–92)	76 (64–86)	<0.001
Antihypertensive therapy, N (%) ^1,2^	18 (42.9)	97 (77.6)	<0.001
Angiotensin-Converting Enzyme inhibitors N (%)	15 (42.8)	70 (35.2)	
Angiotensin Receptor Blockers, N (%)	0 (0)	15 (7.5)	
Beta blockers, N (%)	8 (22.8)	49 (24.6)	
Calcium channel blockers, N (%)	6 (17.1)	32 (16.1)	
Diuretics, N (%)	6 (17.1)	33 (16.6)	
Antihyperlipemic therapy (Statins), N (%) ^1^	8 (19.0)	26 (20.8)	0.807
Type 2 diabetes, N (%) ^1^	6 (14.3)	35 (28.0)	0.074
Smoking status, N (%)^1^	15 (35.7)	24 (19.2)	0.053
Alcohol consumption, N (%) ^1^	15 (35.7)	38 (30.4)	0.473
Physical activity, N (%) ^1^	16 (38.1)	39 (31.2)	0.515
Glucose, mmol/L	5.4 (4.7–5.9)	5.9 (5.3–6.6)	<0.001
Total proteins, g/L ^3^	72 ± 4	72 ± 4	0.953
Albumin, g/L	45 (43–48)	44 (42–47)	0.161
Total bilirubin, μmol/L	11.6 (8.0–14.7)	11.6 (9.2–15.6)	0.472
Direct bilirubin, μmol/L	2.1 (1.5–2.9)	2.3 (1.8–2.8)	0.127
CRP, mg/L	1.9 (0.8–5.1)	2.7 (1.5–6.0)	0.001
Urea, mmol/L	5.5 (4.1–6.4)	5.9 (4.8–7.1)	0.020
Creatinine, μmol/L ^3^	78 (67–91)	79 (68–93)	0.494
Uric acid, μmol/L ^3^	330 ± 81	399 ± 106	<0.001

Data are presented as median and interquartile range. ^1^ Categorical variables are presented as absolute and relative frequencies. ^2^ Many patients in both groups received multiple antihypertensive medications; therefore, the total number of patients receiving antihypertensive therapy differs from the number of patients receiving each therapy. ^3^ Data are presented as mean ± standard deviation. CRP was adjusted for age and BMI (continuous variables) and sex, antihypertensive therapy, antihyperlipemic therapy, and type 2 diabetes (categorical variables).

**Table 2:** Redox status and inflammatory biomarkers of the studied groups.

	Non-OSA Patients	OSA Patients	*p*
AOPP, μmol/L	37.6 (50.3–75.9)	50.0 (52.1–73.6)	0.254
tSH groups, μmol/L	0.461 (0.291–0.587)	0.443 (0.312–0.562)	0.748
SOD, U/L	88 (76–133)	100 (83–131)	0.981
O_2_^.−^, μmol/L ^1^	38.80 (33.62–43.97)	39.08 (36.34–41.09)	0.928
TBARS, μmol/L	2.96 (2.52–3.19)	2.96 (2.67–3.26)	0.879
PAB, HKU	76 (63–93)	73 (62–88)	0.454
IMA, g/L ^1^	0.61 (0.52–0.70)	0.58 (0.53–0.63)	0.079
TOS, μmol/L	24 (12–33)	26 (17–30)	0.631
TAS, μmol/L	1242 (1142–1357)	1311 (1209–1389)	0.001
OSI	1.82 (0.92–2.55)	1.96 (1.18–2.41)	0.662
sRAGE, pg/mL	1242 (936–1584)	940 (648–1446)	0.001
Normalized flRAGE mRNA	1.16 (0.90–1.78)	1.04 (0.84–1.39)	0.665
Normalized TGF-β1 mRNA	1.36 (1.03–1.63)	1.23 (1.02–1.49)	0.335

Data are presented as median (interquartile range). ^1^ Data are presented as adjusted mean (95% CI). Data are adjusted for age, BMI, CRP, uric acid (continuous variables), sex, antihypertensive therapy, antihyperlipemic therapy, and type 2 diabetes (categorical variables), except for TAS, where uric acid was removed from the adjusted factors as it is part of the non-enzymatic defence that contributes to TAS. TAS was adjusted for age, BMI, CRP (continuous variables), sex, antihypertensive therapy, antihyperlipemic therapy, and type 2 diabetes (categorical variables).

**Table 3:** Spearman correlation analysis of polysomnographic data, clinical, inflammatory, and redox status markers in all participants of the studied groups.

	AHI, n/h	ODI, n/h	Average SaO_2_, %	Minimal SaO_2_, %
Age, years	0.200 ^2^	0.205 ^2^	−0.271 ^1^	−0.278 ^1^
BMI, kg/m^2^	0.506 ^1^	0.554 ^1^	−0.559 ^1^	−0.587 ^1^
Glucose, mmol/L	0.347 ^1^	0.311 ^1^	−0.284 ^1^	−0.285 ^1^
Total proteins, g/L	0.103	0.104	−0.111	−0.159 ^3^
Albumin, g/L	−0.016	−0.049	0.140	0.098
Total bilirubin, μmol/L	−0.013	−0.040	0.025	0.014
Direct bilirubin, μmol/L	0.031	0.016	−0.013	−0.008
CRP, mg/L	0.215 ^2^	0.274 ^1^	−0.276 ^1^	−0.273 ^1^
Urea, mmol/L	0.181 ^3^	0.193 ^3^	−0.189 ^3^	−0.217 ^2^
Creatinine, μmol/L	0.138	0.110	−0.110	−0.137
Uric acid, μmol/L	0.260 ^2^	0.387 ^1^	−0.391 ^1^	−0.421 ^1^
AOPP, μmol/L	0.160 ^3^	0.157 ^3^	−0.209 ^2^	−0.240 ^2^
tSH groups, μmol/L	0.142	0.130	−0.105	−0.083
SOD, U/L	0.085	0.057	−0.082	−0.128
O_2_^.−^, μmol/L	−0.104	−0.120	0.167 ^3^	0.154 ^3^
TBARS, μmol/L	0.070	−0.066	−0.089	−0.101
PAB, HKU	−0.048	−0.025	−0.034	−0.038
IMA, g/L	0.151	0.129	−0.092	−0.128
TOS, μmol/L	0.199 ^3^	0.186 ^3^	−0.178 ^3^	−0.213 ^2^
TAS, μmol/L	0.085	0.122	−0.157 ^3^	−0.172 ^3^
OSI	0.159 ^3^	0.143	−0.133	−0.158 ^3^
sRAGE, pg/mL	−0.305 ^1^	−0.313	0.352 ^1^	0.266 ^2^
Normalized flRAGE mRNA	−0.096	−0.102	0.192 ^3^	0.155 ^3^
Normalized TGF-β1 mRNA	−0.028	−0.019	0.062	0.037

^1^ *p* < 0.001; ^2^ *p* < 0.01; ^3^ *p* < 0.05

**Table 4:** Factors extracted by PCA with percentage of variability and variables’ loadings.

Factors	Variables (Loadings)	Factor Variability
Redox status-related factor	TOS (0.920) SOD (0.749) AOPP (0.740) IMA (0.687) TAS (−0.652) tSH groups (0.582)	27%
Proinflammatory genes-related factor	Normalized TGF-β1 mRNA (0.751) Normalized flRAGE mRNA (0.719)	13%
sRAGE-ROS-related factor	sRAGE (0.761) O_2_^.−^ (0.635)	11%
Pro-oxidant-related factor	PAB (0.817) TBARS (0.599)	11%

**Table 5:** Univariable binary logistic regression analysis of PCA factors in the prediction of OSA.

Predictors	B (SE)	Wald	Unadjusted OR (95%CI)	*p*
Redox status-related factor	−0.010 (0.198)	0.002	0.990 (0.672–1.461)	0.961
Proinflammatory genes-related factor	−0.286 (0.187)	2.335	0.751 (0.520–1.084)	0.126
sRAGE-ROS-related factor	−0.566 (0.217)	6.801	0.568 (0.371–0.869)	0.009
Pro-oxidant-related factor	−0.144 (0.192)	0.567	0.866 (0.594–1.260)	0.451

**Table 6:** Demographic, clinical, and laboratory characteristics in OSA patients according to apnea severity.

	Mild OSA N = 29	Moderate OSA N = 31	Severe OSA N = 65	*p*
Male, N (%) ^1^	17 (58.5%)	20 (64.5%)	52 (80%)	0.068
Age, years ^3^	59 ± 11	61 ± 10	57 ± 13	0.213
BMI, kg/m^2^	28 (26–32)	31 (28–32)	35 (32–41) ^a^*^,b^*	<0.001
SBP, mm Hg	130 (130–140)	130 (120–150)	135 (130–145)	0.516
DBP, mm Hg	80 (80–90)	90 (80–100)	85 (80–90)	0.097
AHI, n/h	9.6 (8.1–12.1)	24.2 (19.4–25.6) ^a^*	54.7 (46.4–68.5) ^a^*^,b^*	<0.001
ODI, n/h	10.3 (7.3–12.7)	22.9 (17.9–28.2) ^a^*	64.4 (51.2–73.6) ^a^*^,b^*	<0.001
Average SaO_2_, %	95 (94–96)	94 (91–95) ^a^***	90 (87–93) ^a^*^,b^*	<0.001
Minimal SaO_2_, %	86 (84–88)	80 (71–86) ^a^**	68 (61–76) ^a^*^,b^*	<0.001
Antihypertensive therapy, N (%) ^1,2^	22 (75.9)	23 (74.2)	52 (80.0)	0.789
Angiotensin-Converting Enzyme inhibitors N (%)	15 (34.9)	16 (31.4)	39 (46.4)	
Angiotensin Receptor Blockers, N (%)	4 (9.3)	5 (9.80)	6 (7.10)	
Beta blockers, N (%)	12 (27.9)	13 (25.5)	24 (28.6)	
Calcium channel blockers, N (%)	6 (14.0)	8 (15.7)	18 (21.4)	
Diuretics, N (%)	6 (14.0)	9 (17.6)	18 (21.4)	
Antihyperlipemic therapy (Statins), N (%) ^1^	4 (13.8)	6 (19.4)	16 (24.6)	0.478
Type 2 diabetes, N (%) ^1^	4 (13.8)	7 (22.6)	24 (36.9)	0.052
Smoking status, N (%) ^1^	3 (10.3)	6 (19.9)	15 (23.1)	0.507
Alcohol consumption, N (%) ^1^	6 (20.7)	10 (32.3)	22 (33.8)	0.547
Physical activity, N (%) ^1^	11 (37.9)	9 (29.0)	19 (29.2)	0.393
Glucose, mmol/L	5.6 (5.2–6.0)	6.0 (5.3–6.5)	6.1 (5.6–7.0) ^a^**	0.019
Total proteins, g/L ^3^	72 ± 4	72 ± 4	73 ± 4	0.207
Albumin, g/L ^3^	45 ± 4	43 ± 3	45 ± 3	0.063
Total bilirubin, μmol/L	12.6 (9.6–15.9)	11.5 (10.1–17.8)	11.6 (9.1–14.3)	0.649
Direct bilirubin, μmol/L	2.4 (2.0–3.4)	2.3 (1.7–2.9)	2.3 (1.8–2.8)	0.433
CRP, mg/L	2.2 (1.1–3.9)	2.6 (1.5–5.9)	3.1 (1.6–6.8)	0.364
Urea, mmol/L	5.5 (4.9–6.6)	5.3 (4.6–6.6)	6.2 (5.0–7.0)	0.230
Creatinine, μmol/L	76 (60–87)	74 (62–93)	85 (80–90) ^a^***	0.055
Uric acid, μmol/L	329 (275–454)	360 (303–461)	422 (366–480) ^a^**^,b^***	0.005

Data are presented as median (interquartile range). ^1^ Categorical variables are presented as absolute and relative frequencies. ^2^ Many patients in all three groups received multiple antihypertensive medications; therefore, the total number of patients receiving antihypertensive therapy differs from the number of patients receiving each therapy. ^3^ Data are presented as mean ± standard deviation. CRP was adjusted for age, BMI (continuous variables), sex, antihypertensive therapy, antihyperlipemic therapy, and type 2 diabetes (categorical variables). a—significantly different from Mild OSA. b—significantly different from Moderate OSA. * *p* < 0.001; ** *p* < 0.01, *** *p* < 0.05.

**Table 7:** Redox status and inflammatory biomarkers in OSA patients according to apnea severity.

	Mild OSA N = 29	Moderate OSA N = 31	Severe OSA N = 65	*p*
AOPP, μmol/L	53.7 (49.9–60.4)	58.3 (52.8–71.3)	62.7 (53.2–75.6) ^a,b^*	0.001
tSH groups, μmol/L	0.418 (0.284–0.502)	0.424 (0.289–0.502)	0.491 (0.357–0.633) ^a,b^*	0.007
SOD, U/L ^1^	101 (89–113)	101 (90–113)	107 (99–116)	0.631
O_2_^.−^, μmol/L	40 (32–45)	31 (25–44)	40 (29–53)	0.733
TBARS, μmol/L	3.04 (2.74–3.26)	2.89 (2.67–3.22)	2.96 (2.67–3.26)	0.688
PAB, HKU	72 (62–91)	77 (63–92)	70 (61–86)	0.504
IMA, g/L	0.50 (0.34–0.65)	0.53 (0.37–0.78)	0.64 (0.43–0.79)	0.470
TOS, μmol/L	19 (11–26)	26 (18–33) ^a^*	27 (21–33) ^a^*	0.004
TAS, μmol/L ^1^	1383 (1306–1460)	1289 (1245–1333)	1291 (1259–1323)	0.058
OSI	1.26 (0.76–2.06)	1.99 (1.41–2.51)	2.09 (1.54–2.66) ^a,b^**	0.012
sRAGE, pg/mL	1015 (770–1611)	1042 (710–1592)	820 (546–1122) ^a^*^,b^**	0.006
Normalized flRAGE mRNA	1.15 (0.85–1.52)	1.03 (0.85–1.41)	1.04 (0.80–1.35)	0.996
Normalized TGF-β1 mRNA	1.18 (1.03–1.46)	1.09 (0.91–1.34)	1.26 (1.07–1.52) ^b^*	0.010

Data are presented as median (interquartile range). ^1^ Data are presented as adjusted mean (95%CI). Data are adjusted for age, BMI, CRP, uric acid (continuous variables), sex, antihypertensive therapy, antihyperlipemic therapy, and type 2 diabetes (categorical variables), except for TAS, where uric acid was removed from the adjusted factors as it is part of the non-enzymatic defence that contributes to TAS. TAS was adjusted for age, BMI, CRP (continuous variables), sex, antihypertensive therapy, antihyperlipemic therapy, and type 2 diabetes (categorical variables). a-significantly different from mild OSA. b-significantly different from moderate OSA. * *p* < 0.01; ** *p* < 0.05.

**Table 8:** Spearman correlation coefficients of polysomnographic data, clinical, and redox status markers in OSA patients.

	AHI, n/h	ODI, n/h	Average SaO_2_, %	Minimal SaO_2_, %
Age, years	−0.114	−0.086	−0.037	−0.067
BMI, kg/m^2^	0.461 ^1^	0.503 ^1^	0.518 ^1^	0.584 ^1^
Glucose, mmol/L	0.266 ^2^	0.219 ^3^	−0.152	−0.206 ^3^
Total proteins, g/L	0.172	0.156	−0.186 ^3^	−0.237 ^2^
Albumin, g/L	0.121	0.077	0.066	0.040
Total bilirubin, μmol/L	−0.137	−0.192 ^3^	0.107	0.119
Direct bilirubin, μmol/L	−0.145	−0.177 ^3^	0.140	0.162
CRP, mg/L	0.151	0.221 ^3^	−0.239 ^2^	−0.269 ^2^
Urea, mmol/L	0.079	0.095	−0.130	−0.140
Creatinine, μmol/L	0.145	0.105	−0.160	−0.145
Uric acid, μmol/L	0.235 ^2^	0.273 ^2^	−0.371 ^1^	−0.395 ^1^
AOPP, μmol/L	0.270 ^2^	0.259 ^2^	−0.246 ^2^	−0.385 ^1^
tSH groups, μmol/L	0.255 ^2^	0.229 ^3^	−0.154	−0.116
SOD, U/L	0.122	0.071	−0.031	−0.106
O_2_^.−^, μmol/L	0.069	0.008	0.069	0.039
TBARS, μmol/L	0.022	0.011	−0.083	−0.086
PAB, HKU	0.017	0.062	−0.046	−0.114
IMA, g/L	0.245 ^2^	0.224 ^3^	−0.150	−0.176
TOS, μmol/L	0.308 ^2^	0.278 ^2^	−0.162	−0.261 ^2^
TAS, μmol/L	−0.055	−0.011	−0.167	−0.154
OSI	0.272 ^1^	0.241 ^2^	−0.106	−0.191 ^2^
sRAGE, pg/mL	−0.283 ^2^	−0.276 ^2^	0.297 ^2^	0.217 ^2^
Normalized RAGE mRNA	−0.031	−0.031	0.201 ^3^	0.093
Normalized TGF-β1 mRNA	0.109	0.102	−0.025	−0.065

^1^*p* < 0.001; ^2^ *p* < 0.01; ^3^ *p* < 0.05.

**Table 9:** Univariate and multivariate ordinal logistic regression for the associations of redox status markers and AHI in OSA patients.

Predictors	β (SE)	Wald	Unadjusted OR (95%CI)	*p*
TOS, μmol/L	0.041 (0.015)	7.877	1.042 (1.012–1.071)	0.005
OSI	0.389 (0.160)	5.923	1.475 (1.079–2.017)	0.015
sRAGE, pg/mL	−0.001 (0)	4.690	0.579 (0.352–0.949)	0.030
AOPP, μmol/L	0.028 (0.012)	5.849	1.028 (1.005–1.052)	0.016
IMA, g/L	1.636 (0.758)	4.657	5.134 (1.162–22.692)	0.031
tSH groups	1.787 (0.816)	4.791	5.972 (1.206–29.577)	0.029
**Models**	**β (SE)**	**Wald**	**Adjusted OR (95%CI)**	***p***
TOS, μmol/L	0.037 (0.017)	4.509	1.037 (1.003–1.073)	0.034
OSI	0.319 (0.181)	3.099	1.375 (0.965–1.926)	0.078
sRAGE, pg/mL	0 (0)	1.961	0.691 (0.412–0.691	0.161
AOPP, μmol/L	0.020 (0.012)	2.912	1.020 (0.997–0.967)	0.088
IMA, g/L	1.291 (0.733)	3.104	3.636 (0.965–15.287)	0.078
tSH groups	1.518 (1.004)	2.258	4.563 (0.638–32.622)	0.131

Besides the redox status marker, each model included uric acid, BMI (continuous variables), type 2 diabetes, smoking status, antihypertensive and antihyperlipemic therapies (categorical variables).

**Table 10:** Univariable ordinal logistic regression analysis of PCA factors in the prediction of OSA severity.

Predictors	β (SE)	Wald	Unadjusted OR (95%CI)	*p*
Redox status-related factor	0.469 (0.215)	4.762	1.598 (1.049–2.435)	0.029
Proinflammatory genes-related factor	0.165 (0.205)	0.645	1.179 (0.788–1.765)	0.422
sRAGE-ROS-related factor	−0.195 (0.184)	1.128	0.822 (0.574–1.179)	0.288
Pro-oxidant-related factor	−0.094 (0.184)	0.608	0.910 (0.634–1.305)	0.608

## Data Availability

The data presented in this study are available upon request from the corresponding author. The data are not publicly available due to privacy and ethical considerations.

## Abbreviations

The following abbreviations are used in this manuscript:

OSA	Obstructive sleep apnea
CIH	Chronic intermittent hypoxemia
ROS	Reactive oxygen species
TOS	Total oxidative status
TBARS	Thiobarbiturate reactive substances
TAS	Total antioxidant status
AOPP	Advanced oxidation protein products
CRP	C-reactive protein
flRAGE	Full-length receptor for advanced glycation end products
sRAGE	Soluble receptor for advanced glycation end products
NF-κB	Nuclear factor kappa B
TGF-β1	Transforming growth factor-beta 1
PCA	Principal component analysis
BMI	Body mass index
AHI	Apnea–hypopnea index
ODI	Oxygen desaturation index
SaO_2_	Oxygen saturation
O_2_^.−^	Superoxide anion radical
CV	Coefficient of variation
tSH	Total protein sulfhydryl
SOD	Superoxide dismutase
PAB	Pro-oxidant-antioxidant balance
IMA	Ischemia-modified albumin
OSI	Oxidative stress index
PBMC	Peripheral blood mononuclear cells
RNA	Ribonucleic acid
mRNA	Messenger ribonucleic acid
VIF	Variance inflation factor
CI	Confidence Interval
OR	Odds ratio
HIF-1α	Hypoxia-inducible factor 1-alpha
ECM	Extracellular matrix
